# Study and Mathematical Model of the Chemical Composition and Structure of the Compound Sb_2_(S_1−x_Se_x_)_3_ Based on a Correlation of Data Obtained Through XRD and XPS Characterization

**DOI:** 10.3390/ma19061072

**Published:** 2026-03-11

**Authors:** Martín López-García, Fabio Chalé-Lara, Eugenio Rodríguez-González, Jesús Roberto González-Castillo, Ana B. López-Oyama

**Affiliations:** 1 Instituto Politécnico Nacional, Centro de Investigación en Ciencia Aplicada y Tecnología Avanzada, Unidad Altamira (CICATA-UA), Km 14.5 Carretera Tampico-Puerto Industrial Altamira, Altamira 89600, Tamaulipas, Mexico; fabio_chale@yahoo.com (F.C.-L.); eugenior62@gmail.com (E.R.-G.); 2 Instituto Politécnico Nacional, Escuela Superior de Física y Matemáticas (ESFM), Av. Instituto Politécnico Nacional S/N, Edif. 9 Unidad Profesional Adolfo López Mateos Col. San Pedro Zacatenco, Gustavo A. Madero, Ciudad de México 07738, Mexico; jrgonzalezc@ipn.mx; 3 Departamento de Investigación en Física (DIFUS), Universidad de Sonora, Blvd. Transversal S/N, Hermosillo 83000, Sonora, Mexico; ablopezoy@secihti.mx; 4 Secihti-DIFUS, Universidad de Sonora, Blvd. Transversal S/N, Hermosillo 83000, Sonora, Mexico

**Keywords:** antimony sulfoselenide, stoichiometric, mathematical model

## Abstract

In this work, a study of the chemical composition of the compound Sb_2_(S_1−x_Se_x_)_3_ used in thin-film solar cell fabrication, based on correlating data obtained from XRD and XPS analyses, is presented. This approach enables us to propose a mathematical expression for evaluating stoichiometric variations in the material, showing how the variable x evolves as a function of the diffraction angle 2θ. To establish this model, we analyzed the most intense diffraction peak, corresponding to the (221) plane. To validate the proposed method, a series of Sb_2_(S_1−x_Se_x_)_3_ thin films with different compositions were synthesized using RF-magnetron sputtering followed by conventional heat treatments in a controlled-atmosphere reaction furnace. The XRD results reveal a systematic 2θ shift in the crystalline diffraction peaks toward the positions of the binary precursor phases—from Sb_2_Se_3_ to Sb_2_S_3_—caused by the increased sulfur content during synthesis. XPS measurements confirm the presence of Sb, Se, and S, and high-resolution spectra indicate a decrease in selenium content as the sulfur fraction increases. These results allowed us to elucidate the stoichiometric behavior of antimony sulfoselenide Sb_2_(S_1−x_Se_x_)_3_ using trend curves fitted to the characterization data.

## 1. Introduction

We are currently in the research phase of developing second-generation solar cells, where several semiconductor compounds are competing to become the leading materials for this new technology. Each candidate seeks to demonstrate high performance and strong commercial potential while reducing or eliminating known limitations. For instance, CdTe (cadmium telluride) contains cadmium, a highly toxic element, and Cu_2_InGa(S,Se)_4_ (a chalcopyrite-based material) depends on indium (In), a scarce element in the Earth’s crust [1]. Cu_2_ZnSn(S,Se)_4_ (kesterite) materials have seen limited efficiency progress over the past several years, and their complex, multielement composition inherently promotes the formation of undesirable secondary phases [2]. ABO_3_ and ABX_3_ perovskites (e.g., CaTiO_3_ and CH_3_NH_3_PbI_3_), on the other hand, deliver high performance but are limited by toxicity and poor chemical stability [3].

Antimony chalcogenides are considered newly emerging compounds, and for this reason, they are sometimes excluded from the classification of materials used for research into second-generation solar cells. However, they meet all the characteristics required to be included in this list [4,5,6,7]. The ternary compound antimony sulfo-selenide Sb_2_(S,Se)_3_ has so far managed to outperform the binary compounds Sb_2_Se_3_ and Sb_2_S_3_ in terms of efficiency. In the work by Xiaomin Wang et al., 2020, a certified efficiency of 10.5% was achieved, using the hydrothermal process for film deposition, with the reagents antimony potassium tartrate (APT), sodium thiosulfate (Na_2_S_2_O_3_, STS) and selenourea (SU) as sources of antimony, sulfur and selenium, in addition to ethylenediaminetetraacetic acid (EDT) to regulate the reaction system [8]. Subsequently, Yuqui Zhao et al. 2021, also using the hydrothermal process for film deposition, in addition to a heat treatment based on a solution with alkali metal fluorides, managed to achieve the certified efficiency record of 10.7%, which is still maintained for antimony chalcogenides [9].

**Figure 1:**
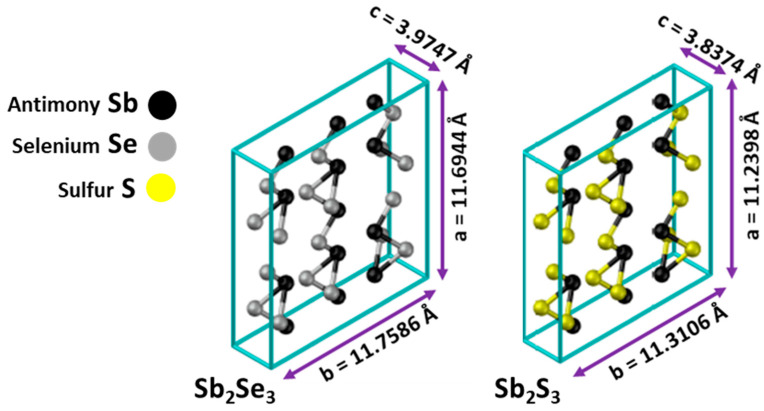
Crystal structures of Sb_2_Se_3_ and Sb_2_S_3_ [11].

Both antimony selenide Sb_2_Se_3_ and antimony sulfide Sb_2_S_3_ are precursor compounds of antimony sulfo-selenide Sb_2_(S, Se)_3_, having almost isomorphic crystalline structures of the orthorhombic type, forming one-dimensional 1d nanostructures in the form of chains (nanoribbons) [10], as shown in Figure 1.

The most outstanding properties of the ternary compound antimony sulfo-selenide Sb_2_(S, Se)_3_ are the following: it is a good light absorber, it has a tunable band gap between 1.1 eV (Sb_2_Se_3_) and 1.7 eV (Sb_2_S_3_) (aspect related to composition) [12], it has a relatively high absorption coefficient ~10^4^–10^5^ cm^−1^, the elements that make up the material are abundant in the Earth’s crust, and the compound itself is of low toxicity and with good chemical stability. In addition, the nanostructures (1d) in the form of chains (nanoribbons) of Sb_2_(S, Se)_3_ favor the transport of charges in that direction (perpendicular to the films) [13,14]. Most of these properties depend on the proportions of sulfur and selenium in the compound through the S/(S+Se) ratio. The chemical equations Sb_2_(S_1−x_Se_x_)_3_ or Sb_2_(S_x_Se_1−x_)_3_ describe this material in more detail, even at the extremes where Sb_2_Se_3_ and Sb_2_S_3_ are also included when x = 0 and 1, giving rise to ternary compounds very similar to their binary counterparts. As can be seen, knowing the composition of this material is fundamental to understanding its performance as an absorber. This aspect not only influences optical properties (band gap) but also structural aspects that can generate lattice coupling or decoupling when it comes into contact with other materials, decreasing or increasing losses due to recombination processes at the interfaces. Furthermore, the composition can cause significant changes in the energy levels of the material, including the Fermi level, due to charge carrier concentrations that can significantly affect the alignment of energy bands, thus improving its performance. Therefore, having a tool that allows for the rapid and accurate determination of this characteristic represents a significant contribution to the field of solar cells. This article presents a detailed analysis based on the correlation of XPS and XRD spectra, which led to the proposal of a mathematical model (a second-degree polynomial) to determine the composition of the compound Sb_2_(S_1−x_Se_x_)_3_ using only the information obtained from a diffractogram.

## 2. Materials and Methods

### 2.1. Experimental Methodology

The ternary compound Sb_2_(S_1−x_Se_x_)_3_ was synthesized by a two-step procedure: first, Sb_2_Se_3_ thin films (the precursors) were deposited at room temperature using RF-magnetron sputtering system supplied by Angstrom Engineering Inc. (Cambridge, ON, Canada). Subsequently, the third element (sulfur) was incorporated into the material via thermal treatment in a reaction furnace with a controlled atmosphere, where in addition the crystalline phase of the films was induced.

### 2.2. Deposition of Sb_2_Se_3_ Precursor Thin Films

A series of five Sb_2_Se_3_ films were deposited using a 99.99% pure Sb_2_Se_3_ target from Advanced Engineering Materials Co., Ltd. (AEM Deposition), Changsha, China. on glass slide substrates (sodium-lime). The parameters used to process the precursor films were a target for the substrate distance of 80 mm, power of 100 watts, substrate rotation of 60 RPM, argon flow rate of 20 SCCM and a working pressure of 0.63 Pa. The sputtering time was 25 min for all samples. Prior to each deposition, the system was evacuated to a base pressure of around 10^−3^ Pa. Using profilometry, the film thickness was measured at five different points of the sample surface. Values differ by less than 1%, indicating the good uniformity of the films. The average thickness was 1100 nm. Using the thickness, a deposition rate of 44 nm/min was calculated.

### 2.3. Incorporation of Sulfur into Sb_2_Se_3_ Precursor Thin Films

**Figure 2:**
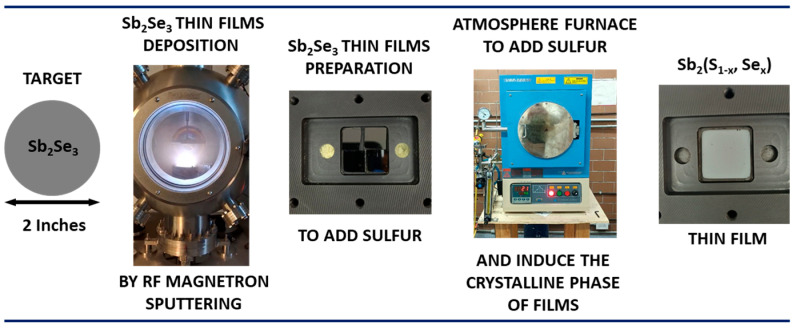
Diagram illustrating the sequential steps of the experimental methodology.

To obtain the ternary antimony sulfo-selenide compound Sb_2_(S,Se)_3_, the precursor films were subjected to a thermal treatment under controlled argon atmosphere. The samples to be treated were placed inside a graphite box with specially designed cavities to accommodate the precursor sample (2.5 × 2.5 cm^2^) and the material to be added (in this experiment, sulfur), as shown in Figure 2. Finally, the box was closed, screwed shut, and placed inside a quartz tube to undergo thermal treatment at the programmed temperature. For the annealing, the temperature was raised from 25 °C (ambient) to 490 °C in 25 min (approx. 18.6 °C/min). The annealing duration was 30 min.

During these heat treatments, different sulfur amounts of 0, 25, 50, 75, and 100 mg were introduced. The initial argon pressure inside the furnace chamber was 75 × 10^3^ Pa, increasing to 101.3 × 10^3^ Pa at the end of the process due to sulfur evaporation.

### 2.4. Characterization

#### 2.4.1. XRD

The crystal structure of the samples was studied by X-ray diffraction (XRD) using a Bruker D8 Advance diffractometer (GmbH, Karlsruhe, Germany) with a Cu Kα radiation source (λ = 1.5406 Å). All samples were analyzed in the Bragg–Brentano (θ-2θ) geometry, with a voltage of 40 kV and a current of 40 mA, in the range 10° ≤ 2θ ≤ 60°, angular step of 0.02°, measurement time 1 s/step and rotating sample.

#### 2.4.2. XPS

XPS measurements were performed on a Thermo Fisher Scientific K-Alpha (Thermo Fisher Scientific, Hillsboro, OR, USA) with a monochromatic Al Kα radiation source, 1486.6 eV. A 180° double-focusing hemispherical analyzer, operated in constant analyzer energy (CAE) mode, was used to measure the kinetic energy of the emitted photoelectrons. Survey and high-resolution spectra were collected using pass energies of 200 and 20 eV, respectively. The Shirley method was used to remove the background from the spectra, and XPSpeak4.1 software was used for processing the XPS signals.

## 3. Results

### 3.1. Structural Characterization of Sb_2_(S_1−x_Se_x_)_3_ Films

**Figure 3:**
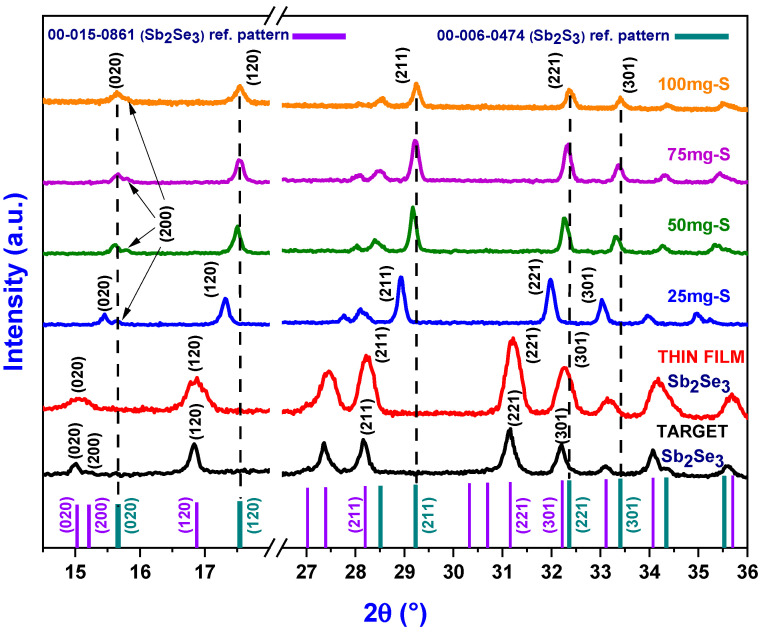
Diffractograms of heat-treated Sb_2_(S_1−x_Se_x_)_3_ films, where 0, 25, 50, 75 and 100 mg of sulfur were added during the treatment. For comparison, the target diffractogram and the reference patterns of both Sb_2_Se_3_ and Sb_2_S_3_ are included in the figure.

Figure 3 shows a comparison of the X-ray diffractograms of the heat-treated films with 0, 25, 50, 75 and 100 mg of sulfur, as well as of the Sb_2_Se_3_ target used in the production of the films. Reference patterns 00-015-0861 (Sb_2_Se_3_, antimony selenide) and 00-006-0474 (Sb_2_S_3_, antimony sulfide) from the ICCD database were used to index the X-ray diffractograms. In the graphs presented, some planes were cropped to better observe the most intense peaks and analyze their behavior.

The X-ray diffractogram exhibits signals at the 2θ positions 15.029°, 15.211°, 16.874°, 27.395°, 28.2°, 31.16°, 32.22°, 33.115°, 34.075° and 35.7°, which correspond to the diffraction planes (020), (200), (120), (230), (211), (221), (301), (311), (240) and (321), respectively. All peaks well match the reference pattern 00-015-0861 associated with the orthorhombic crystal structure of Sb_2_Se_3_.

The diffractogram of the heat-treated Sb_2_Se_3_ film without sulfur (0mg-S) exhibits the same crystallographic reflections as the sputtering target; however, the peaks are slightly broadened and shifted to higher 2θ values. The peak broadening is associated with a decrease in crystallite size. Meanwhile, the shift toward higher angles can be associated with selenium loss during the RF-magnetron sputtering process [15,16].

The signals of highest intensity observed in the diffractograms were located at 2θ positions 28.23° and 31.22°, corresponding to planes (211) and (221), respectively.

Diffractograms further reveal that all signals of the heat-treated films shift to higher 2θ values as the amount of sulfur increases from 0 to 100 mg, indicating that the interplanar distances of the films are experiencing a compression. This angular shift is attributed to the substitution of selenium atoms by sulfur atoms during the thermal treatment process, suggesting the formation of different stoichiometries of antimony sulfo-selenide compounds Sb_2_(S_1−x_Se_x_)_3_ [17].

Specifically, for the diffractogram of the sample heat-treated with 100 mg of sulfur, all planes practically coincide with the pattern associated with Sb_2_S_3_ (pdf: 00-006-0474), suggesting a complete conversion of the Sb_2_Se_3_ precursor film to Sb_2_S_3_.

To demonstrate the evolution in the crystal structure of samples treated with different sulfur quantities, their lattice parameters were calculated using Bragg’s law (Equation (1)) and the equation for the orthorhombic crystal system (Equation (2)), transcribed below:

d_h k l_ = λ/2sen(θ), (1)

1/(d_h k l_)^2^ = h^2^/a^2^ + k^2^/b^2^ + l^2^/c^2^ (2)

where h, k, l are the Miller indices of the Bragg planes (hkl), and d_hkl_ is the interplanar distance. The interplanar distances (d_hkl_) were obtained by Bragg’s law using the signals observed in the of the X-ray diffractograms of the films.

Finally, the edges of the unit cells (a, b, and c) were calculated with the 2θ diffraction angles of the (120), (020), and (002) planes, respectively. In addition, the volumes of these cells were calculated for all films. Results of these calculations are presented in Table 1.

**Figure 4:**
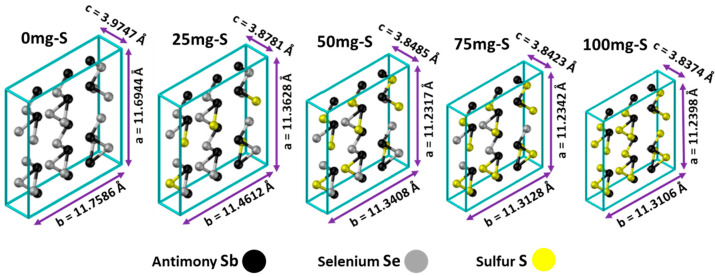
Schematic representation of the compaction process experienced by the antimony sulfo-selenide unit cell Sb_2_(S_1−x_,Se_x_)_3_ dependent on the sulfur amount added during thermal treatment.

Results reveal that the unit cells underwent a contraction process as selenium atoms were gradually replaced by sulfur atoms. The diminution of the unit cell volume occurs because sulfur (at. radius 0.88 Å) atoms are smaller than selenium (at. radius 1.03 Å) atoms. Figure 4 shows a schematic representation of how the unit cell of the compound Sb_2_Se_3_ evolves to Sb_2_S_3_.

**Figure 5:**
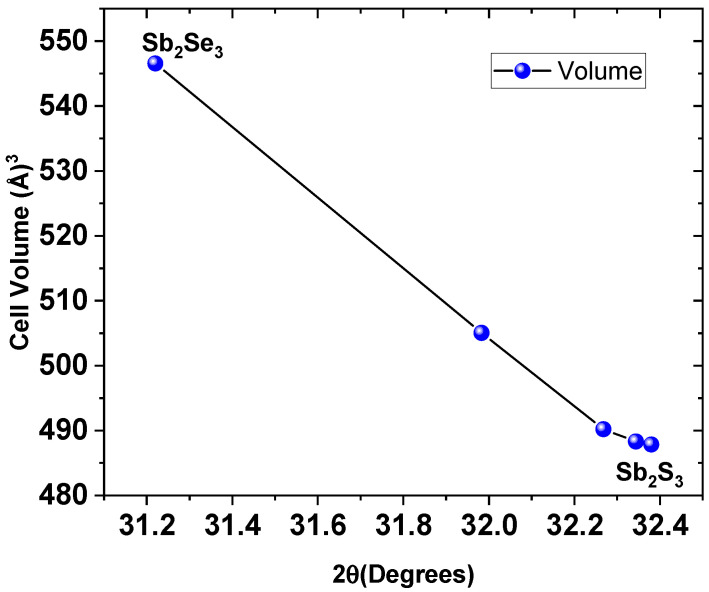
Evolution of the unit cell volume with respect to the 2θ position of the plane (221).

Figure 5 shows the evolution of the unit cell volume of samples as a function of the 2θ position of the (221) plane. Data are taken from Table 1. The (221) plane was selected as the reference because it has 100% relative intensity in the crystallographic patterns employed in this work, making it the most prominent and reliable peak for comparison.

The interpretation of these first results, supported by the data in Table 1, indicate that the sample with 0 milligrams of sulfur (0mg-S) is a film of antimony selenide Sb_2_Se_3_, since sulfur (S) was not added during the heat treatment.

The samples with 25 milligrams of sulfur (25mg-S), 50 milligrams of sulfur (50mg-S) and 75 milligrams of sulfur (75mg-S) correspond to the antimony sulfo-selenide compound Sb_2_(S_1−x_Se_x_)_3_, with different stoichiometries.

Finally, the sample with 100 milligrams of sulfur (100mg-S), corresponds to the antimony sulfo-selenide compound Sb_2_(S_1−x_Se_x_)_3_ also, but with a minimum amount of selenium; therefore, it can be considered as a sample of antimony sulfide Sb_2_S_3_ and related to the reference pattern 00-006-0474.

### 3.2. XPS Compositional Analysis of the Films

**Figure 6:**
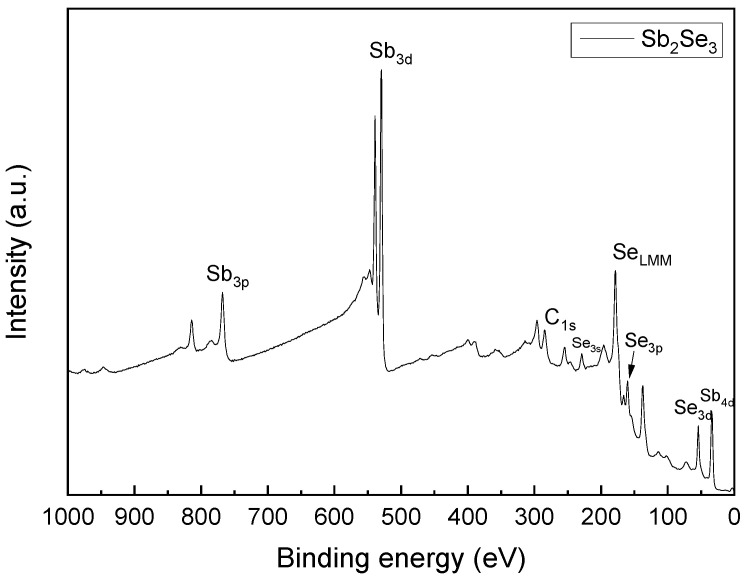
XPS survey spectra for sample 0mg-S of Sb_2_Se_3_.

In Figure 6, a typical survey XPS spectrum of the heat-treated sample with 0 mg of sulfur is presented, where the Sb4d, Se3d, Se3p, Se3p, SeLMM, Se3s, C1s, Sb3d, Se3p and Sb3p signals are observed, confirming the presence of Sb, Se and C. The C1s peak at the position of 284.6 eV was used to correct the peak shift due to charge. The O1s peak is not observed due to overlap with the Sb3d peak, since both signals share the same energy range; as such, the deconvolution of the Sb3d peak was performed, and the different contributions of oxygen and antimony were found.

**Figure 7:**
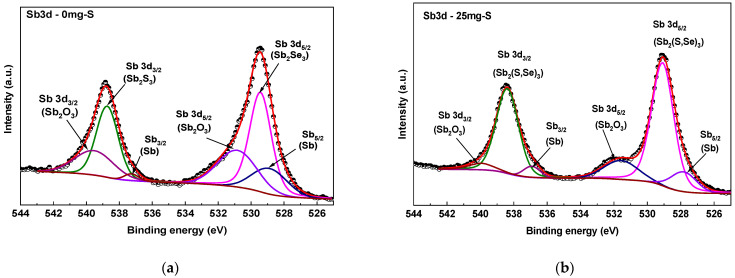

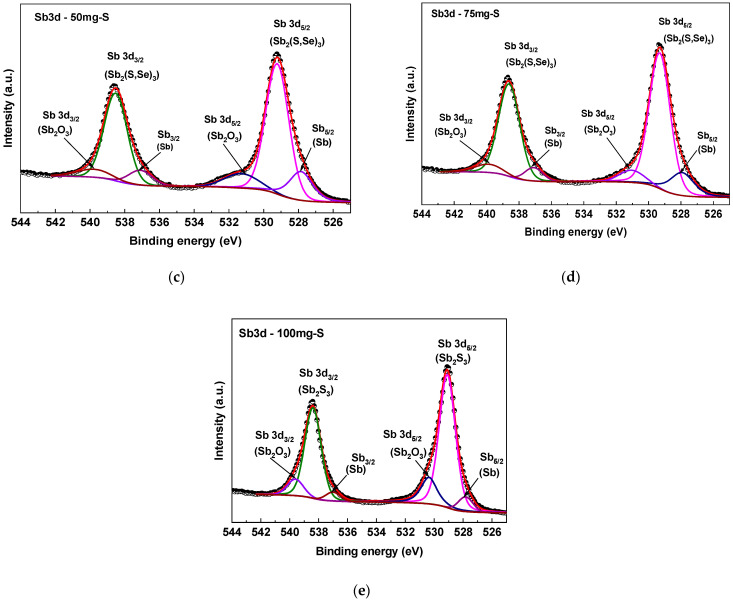
Deconvolution of the XPS Sb3d orbital dependent on the sulfur amount added during the heat treatment: (**a**) 0 mg; (**b**) 25 mg; (**c**) 50 mg; (**d**) 75 mg and (**e**) 100 mg.

Figure 7 depicts the deconvolution of the high-resolution Sb3d spectra for the Sb_2_(S, Se)_3_ films in dependence of the sulfur amount added during the heat treatment. The Sb3d_5/2_ and Sb3d_3/2_ signal areas have a 3:2 ratio in theory, a proportion that can be used in curve fitting of Sb3d XPS spectra [18]. Also, the full width at half maximum (FWHM) was fixed for all peaks used for fitting the spectra.

At 0 mg of sulfur, the spectrum of the Sb3d peak associated with the Sb_2_Se_3_ sample presents the characteristic doublets Sb3d_5/2_ and Sb3d_3/2_ centered at 529.45 and 538.75 eV, respectively, with a separation of 9.3 eV [19]. Additionally, two small shoulders associated with Sb_2_O_3_ were observed, located at 530.67 and 539.96 eV; in a smaller proportion a peak associated with metallic Sb is presented, located at 528.5 and 537.5 eV. For 25, 50 and 75 mg of sulfur in the heat treatment, a similar case occurs, presenting all the same signals as in the first film; however, the peak positions show a slight shift to lower binding energies. For the case of 100 mg of sulfur, the peak positions of Sb3d_5/2_ and Sb3d_3/2_ were 529.09 and 538.39 eV, respectively.

Since the percentage contributions of antimony and oxygen in the spectra cannot be calculated, their areas could not be compared to determine if there was any decrease in antimony in the compounds obtained from the heat treatments. However, no antimony losses have been reported in studies where Sb_2_Se_3_ or Sb_2_S_3_ precursor films were deposited by RF-magnetron sputtering, nor where thin films of Sb_2_(S_1−x_Se_x_)_3_ have been obtained by the thermal evaporation method, which is a very similar process to the heat treatments we have performed in this work.

**Figure 8:**
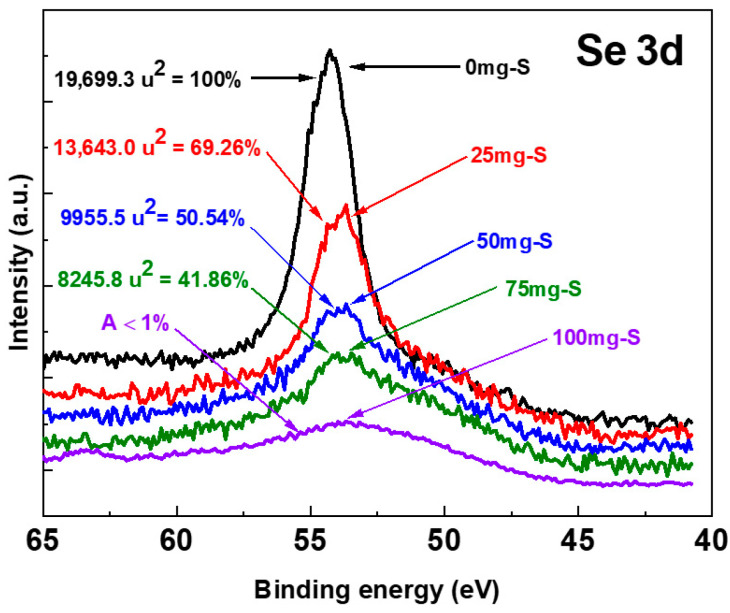
High-resolution XPS spectrum of the Se3d orbital for samples with 0, 25, 50, 75 and 100mg S.

Figure 8 shows the evolution of the Se3d peak for Sb_2_(S, Se)_3_ films deposited and the area percentages of each spectrum, according to the amount of selenium atoms they contain for the heat treatments with 0, 25, 50, 75 and 100 mg of sulfur (S). A gradual decrease in the Se3d peak can be observed as the amount of sulfur increases. These results clearly show the decrease in the selenium content in the films as the amount of sulfur supplied to the samples increases during the heat treatment. The changes in the intensity of the spectra are due to the change in the atomic concentration of Se. It is clear that, in the case of the sample treated with 0 mg of sulfur, the peak area can be attributed solely to selenium atoms.

**Figure 9:**
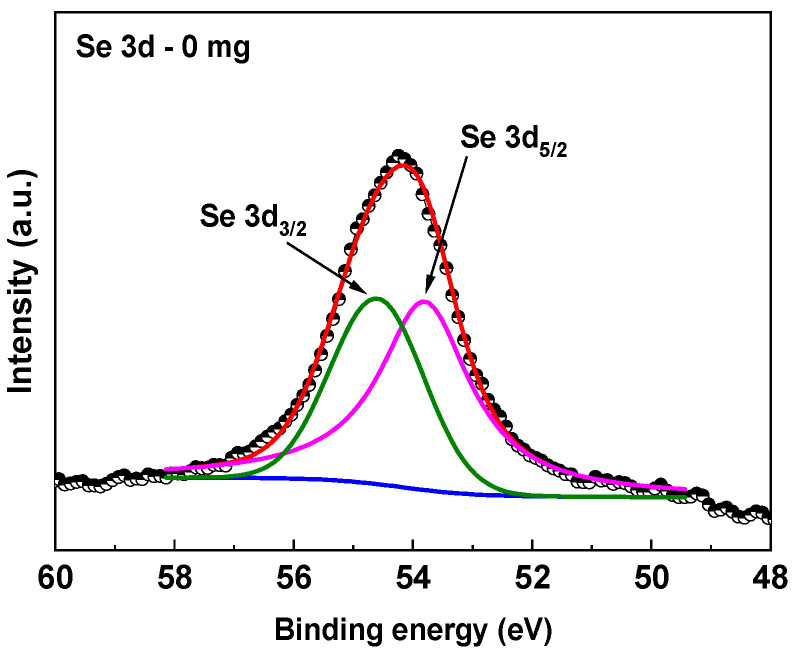
Deconvolution of the XPS Se 3d core-level for sample Sb_2_Se_3_ (0mg S).

An example of the high-resolution spectrum deconvolution of the Se3d peak for heat-treated Sb_2_Se_3_ film with 0 mg of sulfur is shown in Figure 9.

The Se3d_5/2_ and Se3d_3/2_ signal areas have a 3:2 ratio, a proportion which is used in the curve fitting of all Se3d XPS spectra. With the above consideration, the area calculations for the 0, 25, 50 and 75 mg samples of the high-resolution spectra were performed as shown in Figure 8.

For the sample with 100 mg of sulfur, XPS could not quantify selenium, since either no selenium was present or its percentage was very low. Table 2 presents the total area under the Se3d orbital A_Ti_ (i—amount of added sulfur in mg) for each sample, which can be calculated as the sum of the areas under the curve of the 3d_3/2_ and 3d_5/2_ core levels, according to Equation (3):

A_Ti_ = A (3d_3/2_) + A (3d_5/2_) (3)

It is known that the area under the curve for a core-level peak is proportional to the number of atoms of the corresponding element in the sample; several factors must be carefully considered in quantitative analysis, and these are comprehensively addressed in ISO 18118:2024 [20].

This standard establishes a framework that combines mathematical formalism with empirical data. The empirical component is derived from reference samples of known composition, which are used to determine experiment-based relative sensitivity factors (RSFs).

A summary of ISO 18118:2024 Annex A, “Formulae for RSFs,”, provides the equations that enable a more direct and straightforward approach to quantification. It states that, in an electron spectroscopy measurement, quantitative analysis of an unknown, homogeneous sample can be achieved by comparison of peak intensities measured from that sample to the corresponding peak intensities measured from a reference sample with known composition. This intensity ratio is given by Equation (4):

I_i,unk_/I_i,ref_ = (X_i,unk_ × N_unk_ × Q_i,unk_ × R_i,unk_ × λ_i,unk_)/(X_i, ref_ × N_ref_ × Q_i,ref_ × R_i,ref_ × λ_i,ref_) (4)

where X_i_ is the atomic fraction of element i, N is the atomic density, Q_i_ is the correction for elastic electron scattering, R_i_ is the backscattering correction factors for AES (this term is unity for XPS), λ_i_ is the electron inelastic mean free path and the sub-scripts unk and ref denote the values for unknown and reference samples, respectively.

In the present experiment, all samples were analyzed using the same XPS equipment within the same time frame. Under these conditions, the atomic fraction was the only variable, while all other parameters remained constant, since the same element was examined in each case.

This principle applies to any XPS instrument, provided that identical experimental conditions are maintained. Therefore, it is not strictly necessary to perform the measurements simultaneously on the same instrument, as long as the acquisition parameters are equivalent.

The key aspect of this procedure is that the element under investigation (Se) is present in all the samples being compared. Using Equation (4), the number of selenium atoms can be determined as follows (Equation (5)):

Se_atoms_ = [A_Ti_/(19,699.3 u^2^)] × 3 (5)

where 19,699.3 u^2^ is the area under the curve for the core level Se3d of sample without addition of sulfur (Sb_2_Se_3_). This means

19,699.3 u^2^ = 3 selenium atoms

The results obtained using the equation above are presented in Table 3.

To determine the areas under the XPS spectra, a Shirley background was applied to obtain an accurate peak fitting. The quality of the fit was further evaluated using the chi-square (χ^2^) test, which yielded the following values: (AT0, chi squared χ^2^ = 0.95), (AT25, chi squared χ^2^ = 0.875), (AT50, chi squared χ^2^ = 1.044) and (AT75, chi squared χ^2^ = 1.109).

**Figure 10:**
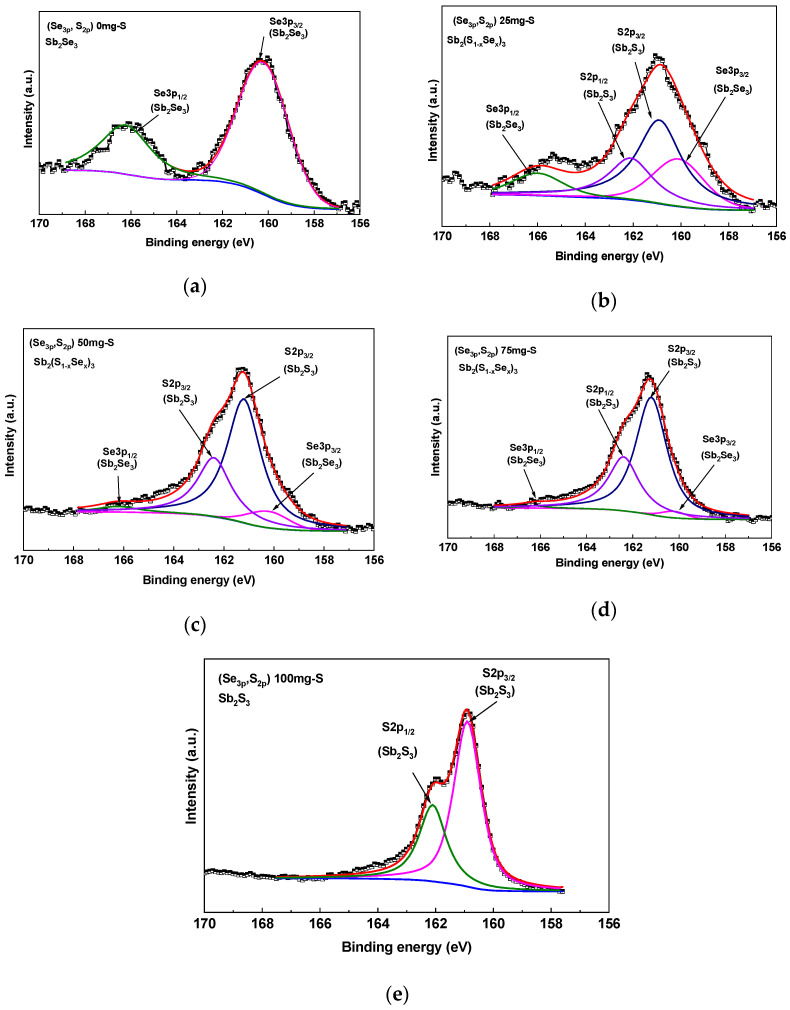
High-resolution spectra of the S2p-Se3p core level for samples with (**a**) 0, (**b**) 25, (**c**) 50, (**d**) 75 and (**e**) 100mg S.

The quantification of S incorporation in Sb_2_Se_3_ samples should also be observed in the S2p peak due to the heat treatment; however, it is difficult because the Se3p and S2p peaks overlap in the interval between 156 and 170 eV. Figure 10 shows deconvolution of the high-resolution Se3p and S2p peaks in Sb_2_Se_3_ samples heat-treated with 0, 25, 50, 75 and 100 mg of sulfur. A clear evolution of the signals is observed. For the 0mg S sample, the spectrum corresponds exclusively to the Se3p region of Sb_2_Se_3_. In this case, the characteristic Se3p_3/2_ and Se3p_1/2_ doublet components are centered at 160.23 and 166.2 eV, respectively, with a separation of 6.0 eV. For the sample with 100 mg of sulfur, a change in the shape of the S2p is observed, with the S 2p_3/2_ and S 2p_1/2_ components centered at 160.9 and 162.1 eV, respectively. In contrast, for the samples treated with 25, 50, and 75 mg of sulfur, four signals were deconvoluted: two attributed to Se 3p_3/2_ and Se 3p_1/2_ at 160.23 and 166.2 eV, respectively, and two corresponding to S 2p_3/2_ and S 2p_1/2_ at 161.2 and 162.3 eV, respectively [21]. The peak areas associated with Se 3p decrease as sulfur is incorporated during the heat treatment, while the S2p peak increases.

## 4. Discussion

### 4.1. Correlation of XRD and XPS Results

**Figure 11:**
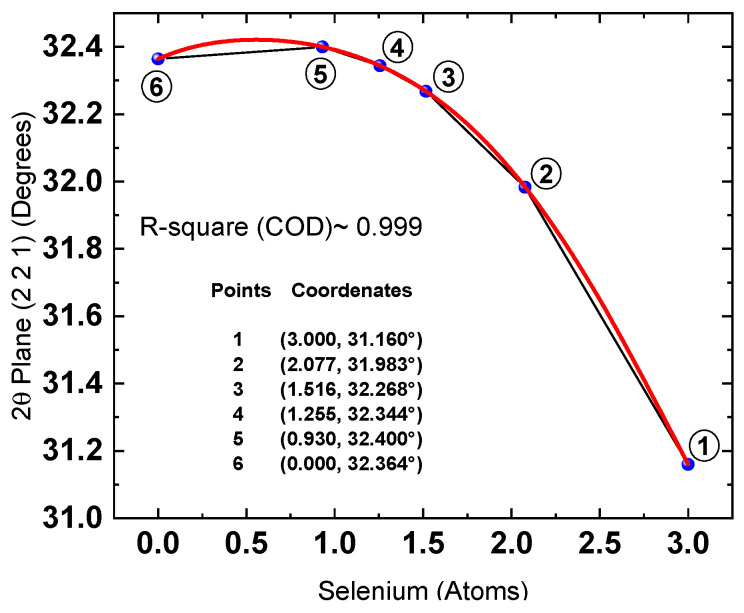
2θ [(221)] plane dependence on the amount of Se atoms obtained from XPS measurements. Red line is a fifth-degree function that best fit the experimental data. Black line is a guide for the eyes. The mathematical function that achieved the best fit to the experimental data, based on the least-squares criterion, was a fifth-degree polynomial, as presented in Equation (6).

Once the XRD and XPS results were evaluated, the data from both techniques were correlated. Figure 11 presents the dependence of the 2θ position of the (221) diffraction plane on the selenium content determined by XPS.

This dependence serves as a calibration curve that enables the estimation of selenium content and, consequently, the compound’s stoichiometry directly from the 2θ position of the XRD signal. Therefore, identifying a mathematical function that accurately describes the 2θ versus Se relationship is essential, as it would allow stoichiometry to be calculated algebraically.

The first point in the graph (3, 31.16°) corresponds to the Sb_2_Se_3_ sputtering target. The diffraction signals observed in its pattern show a strong match with the Sb_2_Se_3_ reference pattern (PDF 00-015-0861).

The second (2.077, 31.983°), third (1.516, 32.268°), and fourth (1.255, 32.344°) points were obtained during the experimental phase, in which samples were thermally treated with the addition of 25, 50, and 75mg of sulfur, respectively. The fifth point (0.93, 32.40°) corresponds to the Sb_2_ (S, Se)_3_ reference pattern (PDF 00-052-1649), for which the reported stoichiometry is Sb_1.99_S_2.07_Se_0.93_.

The selenium content in the sample treated with 100 mg of sulfur could not be quantified by XPS, as it was below the detection limit of the technique. This observation indicates that the resulting compound is nearly stoichiometric Sb_2_S_3_. Therefore, this sample was excluded from the correlation analysis and, instead, the final point (0, 32.364°) from the Sb_2_S_3_ reference pattern (PDF 00-006-0474) was incorporated. In this manner, the trend curve was constructed using three points from reference patterns and three points obtained experimentally.

2θ = 0.01253x^5^ − 0.08494x^4^ + 0.17454x^3^ − 0.30954x^2^ + 0.23457x + 32.364 (6)

where x denotes the number of selenium atoms (0 ≤ x ≤ 3). Although the equation allows the calculation of the 2θ value for a given selenium content, the objective here is the inverse: to determine the selenium concentration directly from the XRD peak position. Therefore, the inverse of the fifth-degree polynomial was obtained. A detailed description of the inversion procedure is provided in the Supplementary Information (Figures S1 and S2). The resulting inverse function is expressed as an infinite power series; however, using only the first nine terms yields sufficiently accurate results. This inverse function was subsequently applied to estimate the number of selenium atoms in the investigated samples, assuming no selenium losses. The calculated values are summarized in Table 4. It is worth pointing out that the number of selenium atoms (stoichiometry) calculated in this study through the correlation between XPS and XRD data is primarily intended for relative compositional comparison rather than for absolute quantitative determination. Furthermore, the polynomial functions fitted to the experimental data represent an empirical interpolation within the measured 2θ range (31.16 ≤ 2θ ≤ 32.364), rather than a generally predictive model.

The maximum intensity point of the (221) reflection for the Sb_2_Se_3_ target used in this study, measured under our experimental conditions, was located at 2θ = 31.163°, whereas the corresponding peak reported in the reference pattern (00-015-0861) appears at 2θ = 31.160°, indicating good instrumental alignment (Δθ ≈ 0.003°). Consequently, the 2θ peak positions of the (221) plane were taken directly from the maximum intensity point in the diffractogram, rather than by fitting a function to the (221) peak profile. However, it is important to emphasize that factors such as residual stress, preferred orientation, or instrumental variability may influence the peak positions and, consequently, any calculations derived from them.

#### 4.1.1. Selenium Losses in Sb_2_Se_3_ Thin Films Caused by the Sputtering Deposition Process

According to the obtained diffractograms, a slight shift in the (221) signal is observed for the Sb_2_Se_3_ film without sulfur addition (2θ = 31.22°), compared to the Sb_2_Se_3_ sputtering target (2θ = 31.16°). Furthermore, noticeable differences in the shape of the XPS spectra between the target and the Sb_2_Se_3_ film were also observed. Both observations suggested compositional differences between the target and the deposited film.

Due to the higher volatility of selenium relative to antimony, selenium vacancies tend to form during the RF sputtering process. As a result, selenium losses can occur during film growth, yielding a composition closer to Sb_2_Se_3−y_ rather than the ideal Sb_2_Se_3_, where y denotes the selenium deficiency. Therefore, the precursor films are expected to grow selenium-deficient, and their calculated stoichiometry (Table 3) must be corrected accordingly.

Using the 2θ position of the (221) reflection for the Sb_2_Se_3−y_ film (2θ = 31.22°) and the previously derived inverse function, the actual selenium content in the sputtered film was calculated as 2.94 atoms per formula unit, corresponding to approximately 2% selenium deficiency. This confirms that the stoichiometry of the Sb_2_Se_3_ films is not fully preserved during sputtering, and the precursor samples exhibit a slight selenium deficit. The selenium content for the remaining samples was recalculated using the same approach and is reported in Table 4.

**Figure 12:**
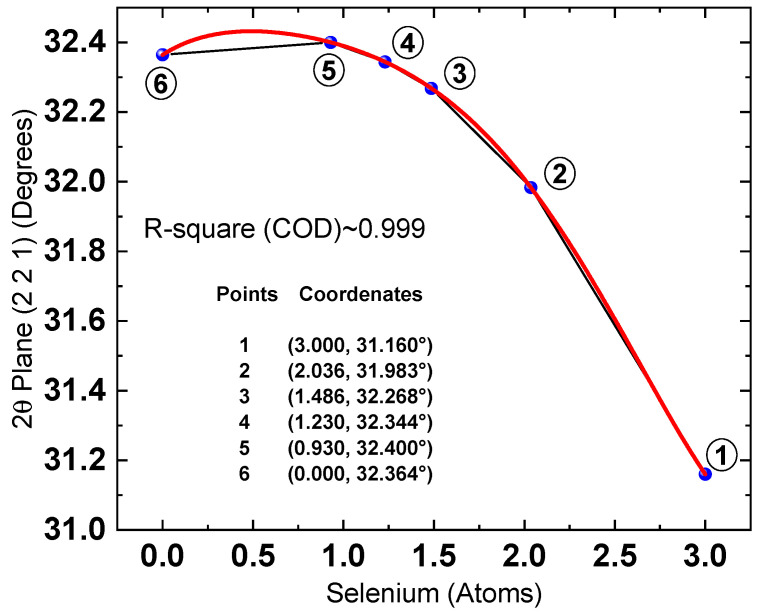
XPS and XRD data correlation. Position 2θ for the plane vs. number of selenium atoms and trend curve (red) fitted to the data obtained by DRX and XPS with 2% of selenium loss.

Using the recalculated selenium contents, the dependence of the 2θ position (221) on the number of Se atoms was replotted, as shown in Figure 12. The mathematical function that best fits the experimental data is presented in Equation (7):

2θ = 0.02559x^5^ − 0.17434x^4^ + 0.39682x^3^ − 0.55781x^2^ + 0.33535x + 32.364 (7)

where x represents the amount of selenium atoms (0 ≤ x ≤ 3).

#### 4.1.2. Trend Curve Using Only the Points of the References Patterns

In the previous section, it was demonstrated that the 2θ (221)–Se dependence (and its inverse function) is highly valuable for determining the Se content from the measured 2θ values, and even for estimating elemental losses during the fabrication process.

**Figure 13:**
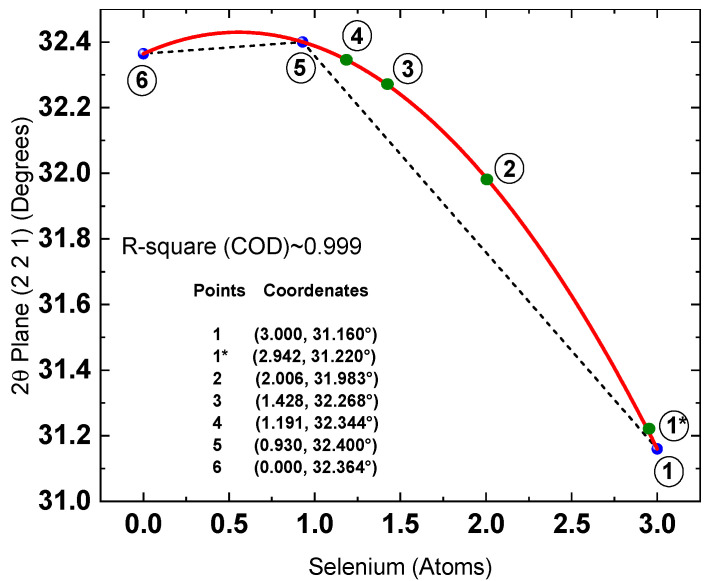
Dependence of the 2θ position of the (221) plane on the number of selenium atoms. Blue points correspond to reference pattern data; the dashed line serves as a guide to the eye, while the red line represents the polynomial fit to these data. Green points indicate the experimental values obtained in this study: Point 1* corresponds to the Sb_2_Se_3_ thin film without sulfur addition, and Points 2, 3, and 4 correspond to samples treated with 25, 50, and 75 mg of sulfur, respectively.

However, deriving this relationship requires correlating XRD and XPS data, which involves substantial experimental effort. As shown in the results obtained so far, the fitted functions accurately describe the (Se, 2θ) data points corresponding to the crystallographic reference patterns. This agreement is essential to ensure the reliability of the developed model. Therefore, the next step was to propose a relationship based solely on the crystallographic reference data for the following compounds: Sb_2_Se_3_ (00-015-0861), Sb_2_S_3_ (00-006-0474), and Sb_2_ (S, Se)_3_ (00-052-1649). The dependence of 2θ on the number of Se atoms derived from these crystallographic sheets (blue points) is shown in Figure 13.

The mathematical function that best fits these data points (with an accuracy of nearly 100%) is a second-degree polynomial, as presented in Equation (8).

2θ = −0.21258x^2^ + 0.23641x + 32.364 (8)

where x represents the number of Se atoms and 2θ is the angular position of the (221) diffraction plane. The results indicate that this function exhibits a maximum at x = 0.556 (2θ_max_ = 32.4297278296°). Although such angular resolution cannot be achieved experimentally, the stoichiometric calculation is highly sensitive to small variations in 2θ, which necessitates the use of extended decimal precision.

The points highlighted in green (1*, 2, 3, and 4) were excluded from the fitting process and subsequently added to Figure 13 following the fitting procedure. The “x” coordinate (2θ value) for these points was directly obtained from the corresponding diffractogram, while the “y” coordinate was derived using the inverse of the second-degree polynomial. The results demonstrate good agreement between the experimental data and the second-order polynomial fit.

The inverse of this second-degree polynomial is presented below. It is worth noting that deriving this inverse function was considerably simpler than in the case of the previously reported fifth-degree function. The variables x_1_ (Equation (9)) and x_2_ (Equation (10)) represent the solutions associated with two distinct 2θ intervals.

x_1_ = [−0.2364 − ([(0.23641)^2^ + 4(−0.21258)(2θ − 32.364)]^0.5^)]/6(−0.21258) (9)

Valid for the interval 31.16° ≤ 2θ ≤ 2θ_max_.

The stoichiometry of the compound within the reported 2θ interval is given by

Sb_2_(S_1−x1_Se_x1_)_3_

x_2_ = [−0.2364 + ([(0.23641)^2^ + 4(−0.21258)(2θ − 32.364)]^0.5^)]/6(−0.21258) (10)

Valid for the interval 2θ_max_ ≤ 2θ ≤ 32.364.

The stoichiometry of the compound within the reported 2θ interval is given by

Sb_2_(S_1−x2_Se_x2_)_3_

In a similar manner to that described in the previous section, the inverse of the quadratic polynomial can be used to determine the selenium content for all samples.

Finally, Table 5 compares the Se content calculated using both the fifth-degree and second-degree polynomial functions, with the aim of identifying which model more accurately describes the variation in x in the stoichiometric equation Sb_2_(S_1−x_Se_x_)_3_.

As observed, the calculations based on the fifth-degree polynomial (assuming a 2% selenium loss) converge toward those obtained using the second-degree polynomial. This confirms that accounting for selenium loss leads to results that more closely match the quadratic model.

## 5. Conclusions

1. In this study, the processing of thin films of the ternary antimony sulfo-selenide compound Sb_2_(S_1−x_Se_x_)_3_ with different stoichiometries is reported. For the synthesis, precursor films of antimony selenide (Sb_2_Se_3_) were deposited by RF-magnetron sputtering and subsequently subjected to thermal treatments in a reaction furnace under a controlled atmosphere to introduce the third element (sulfur) and promote the formation of the crystalline phase of the compound.

2. By varying the amount of sulfur added during the thermal treatment, it was possible to gradually substitute Se atoms with S atoms, yielding films with stoichiometries ranging from antimony selenide (precursor films, 0 mg of S) to antimony sulfide (100 mg of S).

3. By correlating the XRD and XPS data, it was possible to establish the dependence of the 2θ (221) diffraction angle on the Se atomic content in the compound. The experimental data were best fitted using a fifth-degree polynomial, whose inverse function enabled the calculation of the compound’s stoichiometry. This correlation also proved valuable for estimating selenium losses during the RF-magnetron sputtering growth of the precursor films, a phenomenon initially identified through XRD and XPS analysis.

4. Using only the crystallographic data for the three compounds Sb_2_Se_3_S_0_(00-015-0861), Sb_1.99_S_2.07_Se_0.93_(00-052-1649), and Sb_2_S_3_Se_0_(00-006-0474), the 2θ (221)-diffraction angle dependence on the Se atomic content was also determined. The best fit to these data was obtained with a second-degree polynomial. This function also enabled the calculation of the compound’s stoichiometry with an error not exceeding 3% relative to the value determined from the experimental data obtained in this research.

5. Overall, the main contribution of this work is the development of a mathematical model applied to the Sb_2_(S_1−x_Se_x_)_3_ system, which describes the stoichiometric behavior of the compound. Assuming that the data reported in the reference patterns are accurate, and using a second-degree polynomial fit to those data, the stoichiometry of an Sb_2_(S_1−x_Se_x_)_3_ sample can be determined with an error lower than 3%. To the best of our knowledge, such a model has not been previously reported in the literature.

## Figures and Tables

**Table 1:** Data and calculated lattice parameters and unit cell volumes for the different samples.

Parameter	0mg-S	25mg-S	50mg-S	75mg-S	100mg-S
2θ_120_ (°)	16.8640	17.3160	17.5050	17.5390	17.5400
d_120_ (Å)	5.2528	5.1167	5.0619	5.0522	5.0519
a (Å)	11.6944	11.3628	11.2317	11.2342	11.2398
2θ_020_ (°)	15.0560	15.4490	15.6140	15.6530	15.6560
d_020_ (Å)	5.87929	5.73060	5.6704	5.6564	5.6553
b (Å)	11.7586	11.4612	11.3408	11.3128	11.3106
2θ_002_ (°)	45.6080	46.8109	47.1920	47.2730	47.3370
d_002_ (Å)	1.9873	1.9390	1.9243	1.9212	1.9187
c (Å)	3.9747	3.8781	3.8485	3.8423	3.8374
V(Å^3^)	546.5600	505.0500	490.2083	488.3189	487.8440

**Table 2:** Calculated areas for the samples 0mg S, 25mg S, 50mg S and 75mg S. Areas were expressed in square arbitrary units (u^2^).

A_Ti_	Sample	Area (u^2^)
A_T0_	0mg S	19,699.3
A_T25_	25mg S	13,643.0
A_T50_	50mg S	9955.5
A_T75_	75mg S	8245.8

**Table 3:** Calculation of proportion of selenium atoms for the samples 0mg S, 25mg S, 50mg S and 75mg S.

Sample	Area Percentage	Calculation of the Amount of Selenium Atoms	Proportion of Selenium Atoms
0mg S	(19,699.3/19,699.3) × 100 = 100%	(1) × 3	3.000
25mg S	(13,643.0/19,699.3) × 100 = 69.26%	(0.6926) × 3	2.077
50mg S	(9955.5/19,699.3) × 100 = 50.54%	(0.5054) × 3	1.516
75mg S	(8245.8/19,699.3) × 100 = 41.86%	(0.4186) × 3	1.255
100mg S	XPS could not quantify	N/A	≈0

**Table 4:** Recalculation of the Se atoms number for samples 0mg S, 25mg S, 50mg S and 75mg S, considering 0%, and 2% of selenium loss.

Sample	Area (%)	Se Atoms Without Se Loss Sb_2_Se_3_	Recalculation of Se Atoms (Assuming 2% Loss Sb_2_Se_3−0.06_)
0mg S	100	(1) × 3 = 3.000	(1) × 2.942 = 2.942
25mg S	69.26	(0.6926) × 3 = 2.070	(0.6926) × 2.942 = 2.036
50mg S	50.54	(0.5054) × 3 = 1.510	(0.5054) × 2.942 = 1.486
75mg S	41.86	(0.4186) × 3 = 1.250	(0.4186) × 2.942 = 1.230
100mg S	XPS could not quantify	N/A ≈ 0	N/A ≈ 0

**Table 5:** Comparison values between 5th-degree functions and the 2nd-degree function. Point 1 corresponds to the Sb_2_Se_3_ sputtering target. Point 1* corresponds to the Sb_2_Se_3_ thin film without sulfur addition. Points 2, 3, and 4 correspond to samples treated with 25, 50, and 75 mg of sulfur, respectively.

Points	Fifth-Degree Equation (Assuming 0% Se Losses)	Fifth-Degree Equation (Assuming 2% Se Losses)	Second-Degree Equation
1	(3.000, 31.160°)	(3.000, 31.160°)	(3.000, 31.160°)
1*	(2.940, 31.220°)	(2.926, 31.220°)	(2.942, 31.220°)
2	(2.077, 31.983°)	(2.036, 31.983°)	(2.006, 31.983°)
3	(1.516, 32.268°)	(1.486, 32.268°)	(1.428, 32.268°)
4	(1.255, 32.344°)	(1.230, 32.344°)	(1.191, 32.344°)
5	(0.930, 32.400°)	(0.930, 32.400°)	(0.930, 32.400°)
6	(0.000, 32.364°)	(0.000, 32.364°)	(0.000, 32.364°)

## Data Availability

The original contributions presented in this study are included in the article/Supplementary Materials. Further inquiries can be directed to the corresponding author.

## Supplementary Materials

The following supporting information can be downloaded at https://www.mdpi.com/article/10.3390/ma19061072/s1, Figure S1: 2θ vs. selenuim atoms graph. Figure S2: section graph of fifth degree equation and its second degree auxiliary.
