# Supplementary material for: Study and Mathematical Model of the Chemical Composition and Structure of the Compound Sb2(S1−xSex)3 Based on a Correlation of Data Obtained Through XRD and XPS Characterization
Source: Materials (Basel). 2026 Mar 11;19(6):1072. doi: 10.3390/ma19061072 (PMC13027836; doi:10.3390/ma19061072)
Supplement: Supplementary file 1 [file materials-19-01072-s001.zip › materials-4112264-supplementary.pdf]

SUPLEMENTARY

INVERSE FUNCTIONS

Author: Martín López García

Abstract

The following work, shows the obtaining of inverse functions for any function to which the derivative theorem of an inverse function can be applied. Specifically, in this paper the method is applied to find the inverse function of a fifth degree equation. Although normally for equations of "n" degree the attention is focused on their zeros, in this case it will not be like that, because the interest is not only to find specific values, but the set of values that satisfy solution intervals, properly speaking. said the inverse function. The general form of the inverse functions achieved in this methodology is through infinite power series, therefore they require close initial values to converge quickly in the values of the inverse function, however those initial close values are replaced by functions that touch those close values in certain intervals and cause a more complex function to be generated than the already complex power series, however in this way the true inverse functions sought are achieved.

Introduction.

This work was motivated by the need to obtain the inverse function of an equation of the fifth degree, and is conceived through the theorem of the derivative of an inverse function, where the problem of having to already know the inverse function itself is overcome, through implicit differentiation and the construction of an infinite power series. At first, the method could be compared with the Newton-Raphson method, but in this method iterations are not used, in addition to not only searching for the zeros of the functions and without further ado for the moment, we will go directly to the theoretical-deductive part.

Approach:

Be:

$$f(x) = y$$

$$f'(x) = y'$$

$$f^{-1}(x) = \mathit{inverse\ function\ of}\ f(x)$$

$$f^{-1\prime}(x) = \mathit{derivative\ of\ the\ inverse\ function}$$

$$f^{-1\prime}(x) = \frac{1}{f'(x)} = \frac{1}{y'}$$

$$f^{-1\prime}(x) = \frac{1}{f'[f^{-1}(x)]}$$

An example:

$$f(x) = x^2$$

$$f'(x) = 2x$$

$$f^{-1\prime}(x) = \frac{1}{f'(x)} = \frac{1}{2x}$$

$$f^{-1}(x) = \sqrt{x}$$

Then:

$$f^{-1\prime}(x) = \frac{1}{f'[f^{-1}(x)]} = \frac{1}{2(\sqrt{x})} = \frac{1}{2\sqrt{x}}$$

Deduction of inverse functions through power series:

Coefficients:

$$\boldsymbol{C_0} = f^{-1}(x)$$

$$\boldsymbol{C_1} = f^{-1\prime}(x) = \frac{1}{f'[f^{-1}(x)]}$$

Using implicit differentiation:

$$\boldsymbol{C_2} = f^{-1\prime\prime}(x) = \frac{-f''[f^{-1}(x)]}{(f'[f^{-1}(x)])^2} \cdot \left(\frac{1}{f'[f^{-1}(x)]}\right)$$

$$\boldsymbol{C_2} = f^{-1\prime\prime}(x) = \frac{-f''[f^{-1}(x)]}{(f'[f^{-1}(x)])^3}$$

$$\boldsymbol{C_3} = f^{-1\prime\prime\prime}(x) = \frac{-f'''[f^{-1}(x)]}{(f'[f^{-1}(x)])^4} + \frac{3(f''[f^{-1}(x)])^2}{(f'[f^{-1}(x)])^5}$$

Changing the nomenclatura to a friendlier one for easy and more functionally, as will be seen later:

$$\boldsymbol{C_0} = x_c$$

Let $\mathbf{x_c}$ be a value close to the sought value of the inverse function.

$$\boldsymbol{C_1} = \frac{1}{y'}$$

$$\boldsymbol{C_2} = \frac{-y''}{(y')^3}$$

$$\boldsymbol{C_3} = \frac{-y'''}{(y')^4} + \frac{3(y'')^2}{(y')^5}$$

$$\boldsymbol{C_4} = \frac{-y''''}{(y')^5} + \frac{10y'' \cdot y'''}{(y')^6} - \frac{15(y'')^3}{(y')^7}$$

$$\boldsymbol{C_5} = \frac{-y'''''}{(y')^6} + \frac{15y'' \cdot y'''' + 10(y''')^2}{(y')^7} - \frac{105(y'')^2 \cdot y'''}{(y')^8} + \frac{105(y'')^4}{(y')^9}$$

$$\boldsymbol{C_6} = \frac{-y''''''}{(y')^7} + \frac{21y'' \cdot y''''' + 35y''' \cdot y''''}{(y')^8} - \frac{210(y'')^2 \cdot y'''' + 280y'' \cdot (y''')^2}{(y')^9} + \frac{1260(y'')^3 \cdot y'''}{(y')^{10}} - \frac{945(y'')^5}{(y')^{11}}$$

$$\boldsymbol{C_7} = \frac{-y'''''''}{(y')^8} + \frac{28y'' \cdot y'''''' + 56y''' \cdot y''''' + 35(y'''')^2}{(y')^9} - \frac{378(y'')^2 \cdot y''''' + 1260y'' \cdot y''' \cdot y'''' + 280(y''')^3}{(y')^{10}} + \frac{3150(y'')^3 \cdot y'''' + 6300(y'')^2 \cdot (y''')^2}{(y')^{11}} - \frac{17325(y'')^4 \cdot y'''}{(y')^{12}} + \frac{10395(y'')^6}{(y')^{13}}$$

$$\boldsymbol{C_8} = \frac{-y''''''''}{(y')^9} + \frac{36y'' \cdot y''''''' + 84y''' \cdot y'''''' + 126y'''' \cdot y'''''}{(y')^{10}}$$

$$-\frac{630(y'')^2 \cdot y'''''' + 2520y'' \cdot y''' \cdot y''''' + 1575y'' \cdot (y'''')^2 + 2100(y''')^2 \cdot y''''}{(y')^{11}}$$

$$+\frac{6930(y'')^3 \cdot y''''' + 34650(y'')^2 \cdot y''' \cdot y'''' + 15400y'' \cdot (y''')^3}{(y')^{12}}$$

$$-\frac{138600(y'')^3 \cdot (y''')^2 + 51975(y'')^4 \cdot y''''}{(y')^{13}} + \frac{270270(y'')^5 \cdot y'''}{(y')^{14}} - \frac{135135(y'')^7}{(y')^{15}}$$

$$\boldsymbol{C_9} = \frac{-y'''''''''}{(y')^{10}} + \frac{45y'' \cdot y'''''''' + 120y''' \cdot y''''''' + 210y'''' \cdot y'''''' + 126(y''''')^2}{(y')^{11}}$$

$$-\frac{990(y'')^2 \cdot y''''''' + 4620y'' \cdot y''' \cdot y'''''' + 6930y'' \cdot y'''' \cdot y''''' + 4620(y''')^2 \cdot y''''' + 5775y''' \cdot (y'''')^2}{(y')^{12}}$$

$$+\frac{13860(y'')^3 \cdot y'''''' + 83160(y'')^2 \cdot y''' \cdot y''''' + 51975(y'')^2 \cdot (y'''')^2 + 138600y'' \cdot (y''')^2 \cdot y'''' + 15400(y''')^4}{(y')^{13}}$$

$$-\frac{135135(y'')^4 \cdot y''''' + 900900(y'')^3 \cdot y''' \cdot y'''' + 600600(y'')^2 \cdot (y''')^3}{(y')^{14}}$$

$$+\frac{3153150(y'')^4 \cdot (y''')^2 + 945945(y'')^5 \cdot y''''}{(y')^{15}} - \frac{4729725(y'')^6 \cdot y'''}{(y')^{16}}$$

$$+\frac{2027025(y'')^8}{(y')^{17}}$$

Only 10 coefficients have been calculated and no more will be calculated, because the series is infinite and these are enough to show the procedure. Now applying the method to a fifth degree equation we have the following:

$$f(x) = y = ax^5 + bx^4 + cx^3 + dx^2 + ex + f$$

$$f\ (x_c) = y_c = a{x_c}^5 + b{x_c}^4 + c{x_c}^3 + d{x_c}^2 + ex_c + f$$

$$\boldsymbol{y_c} = value\ close\ to\ "y"$$

Then the inverse function is constructed as follows:

$$f^{-1}(x) = x_c\frac{(y-y_c)^0}{0!} + C_1\frac{(y-y_c)^1}{1!} + C_2\frac{(y-y_c)^2}{2!} + C_3\frac{(y-y_c)^3}{3!} + \cdots + C_n\frac{(y-y_c)^n}{n!}$$

$$n = 0 - \infty$$

$$y = ax^5 + bx^4 + cx^3 + dx^2 + ex + f$$

$$y' = 5ax^4 + 4bx^3 + 3cx^2 + 2dx + e$$

$$y'' = 20ax^3 + 12bx^2 + 6cx + 2d$$

$$y''' = 60ax^2 + 24bx + 6c$$

$$y'''' = 120ax + 24b$$

$$y''''' = 120a$$

$$\boldsymbol{C_0} = x_c$$

$$\boldsymbol{C_1} = \frac{1}{y'} = \frac{1}{5a{x_c}^4 + 4b{x_c}^3 + 3c{x_c}^2 + 2dx_c + e}$$

$$\boldsymbol{C_2} = \frac{-y''}{(y')^3} = \frac{-(20a{x_c}^3 + 12b{x_c}^2 + 6cx_c + 2d)}{(5a{x_c}^4 + 4b{x_c}^3 + 3c{x_c}^2 + 2dx_c + e)^3}$$

$$\boldsymbol{C_3} = \frac{-y'''}{(y')^4} + \frac{3(y'')^2}{(y')^5} = \frac{-(60a{x_c}^2 + 24bx_c + 6c)}{(5a{x_c}^4 + 4b{x_c}^3 + 3c{x_c}^2 + 2dx_c + e)^4} + \frac{3(20a{x_c}^3 + 12b{x_c}^2 + 6cx_c + 2d)^2}{(5a{x_c}^4 + 4b{x_c}^3 + 3c{x_c}^2 + 2dx_c + e)^5}$$

$$\boldsymbol{C_4} = \frac{-y''''}{(y')^5} + \frac{10y'' \cdot y'''}{(y')^6} - \frac{15(y'')^3}{(y')^7}$$

$$\boldsymbol{C_4} = \frac{-(120ax_c + 24b)}{(5a{x_c}^4 + 4b{x_c}^3 + 3c{x_c}^2 + 2dx_c + e)^5} + \frac{10(20a{x_c}^3 + 12b{x_c}^2 + 6cx_c + 2d)(60a{x_c}^2 + 24bx_c + 6c)}{(5a{x_c}^4 + 4b{x_c}^3 + 3c{x_c}^2 + 2dx_c + e)^6}$$
$$- \frac{15(20a{x_c}^3 + 12b{x_c}^2 + 6cx_c + 2d)^3}{(5a{x_c}^4 + 4b{x_c}^3 + 3c{x_c}^2 + 2dx_c + e)^7}$$

No more coefficients will be calculated, because these are enough to solve the example of a fifth degree equation that will be used as an example. Therefore the series would be as follows:

$$f^{-1}(x) = x_c + \frac{y - (ax_c{}^5 + bx_c{}^4 + cx_c{}^3 + dx_c{}^2 + ex_c + f)}{5ax_c{}^4 + 4bx_c{}^3 + 3cx_c{}^2 + 2dx_c + e}$$

$$+ \left[\frac{-(20ax_c{}^3 + 12bx_c{}^2 + 6cx_c + 2d)[y - (ax_c{}^5 + bx_c{}^4 + cx_c{}^3 + dx_c{}^2 + ex_c + f)]^2}{2!\,(5ax_c{}^4 + 4bx_c{}^3 + 3cx_c{}^2 + 2dx_c + e)^3}\right]$$

$$+ \left[\frac{-(60ax_c{}^2 + 24bx_c + 6c)}{(5ax_c{}^4 + 4bx_c{}^3 + 3cx_c{}^2 + 2dx_c + e)^4} + \frac{3(20ax_c{}^3 + 12bx_c{}^2 + 6cx_c + 2d)^2}{(5ax_c{}^4 + 4bx_c{}^3 + 3cx_c{}^2 + 2dx_c + e)^5}\right] \frac{[y - (ax_c{}^5 + bx_c{}^4 + cx_c{}^3 + dx_c{}^2 + ex_c + f)]^3}{3!}$$

$$+ \left[\frac{-(120ax_c + 24b)}{(5ax_c{}^4 + 4bx_c{}^3 + 3cx_c{}^2 + 2dx_c + e)^5} + \frac{10(20ax_c{}^3 + 12bx_c{}^2 + 6cx_c + 2d)(60ax_c{}^2 + 24bx_c + 6c)}{(5ax_c{}^4 + 4bx_c{}^3 + 3cx_c{}^2 + 2dx_c + e)^6} - \frac{15(20ax_c{}^3 + 12bx_c{}^2 + 6cx_c + 2d)^3}{(5ax_c{}^4 + 4bx_c{}^3 + 3cx_c{}^2 + 2dx_c + e)^7}\right] \frac{[y - (ax_c{}^5 + bx_c{}^4 + cx_c{}^3 + dx_c{}^2 + ex_c + f)]^4}{4!}$$

Be:

$$\boldsymbol{2\theta = 0.01253x^5 - 0.08494x^4 + 0.17454x^3 - 0.30954x^2 + 0.23457x + 32.364}$$

This equation arose from a stoichiometric calculation based on diffractograms and XPS studies and turned out to be the graph that best fit the points found, in fact it fit 100 percent. Figure 1.

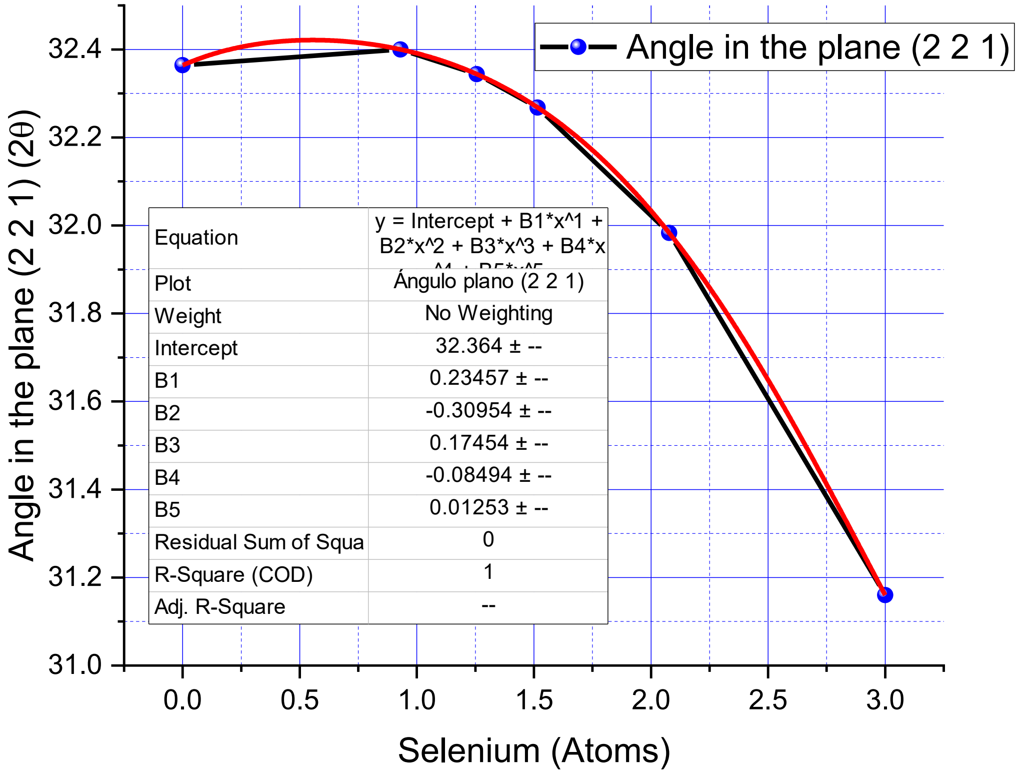

Figure S1: 2θ vs selenium atoms graph.

A problem that is immediately apparent is that it is necessary to find an inverse function for the fifth degree equation, since the data that is initially known in the diffractograms are the 2θ angles out of phase of the (2 2 1) plane. In this way, here the method shown can be applied to find an inverse equation.

The 5-term formula shown above converges for values close to the desired value, that is, to find the value of 3 selenium atoms at 2θ = 31.16°. A value close is 3.226 for "x_c" and finds the value of 3 selenium atoms for 2θ = 31.16°. However the range of 2.5 to 3.3 for x_c gives a very approximate value. A value to find the value of 1.5161 selenium atoms for 2θ = 32.268 is the value x_c = 1.65 which leads to the exact result. As can be seen, the infinite power series with only 5 terms is not satisfactory, unless you have to enter two data to obtain a result (x_c and 2θ), however it is possible to find a function that depends on 2θ for x_c and that passes close to the values that make the series coverge to the desired values. In this way, the following function was found, which meets that objective.

$$x_c = \frac{-0.183388496232336 \pm \sqrt{(0.183388496232336)^2 + 4(-0.203695507874834)(2\theta - 32.364720710102)}}{(2)(-0.203695507874834)}$$

The equation is strict in the number of decimals for very precise calculations; $\mathbf{x_c}$ should go wherever $\mathbf{x_c}$ is in the power series, which will make the inverse function horrendous, however that is the inverse function that it fulfills in the desired interval for the fifth degree equation. There is an Excel program made by me, where you only enter the value of 2θ and it gives the value of the selenium atoms for that angle. Another more precise calculation was carried out with 10 terms of the series, where a program was also created in Excel and with the following second degree function:

$$(x - 0.55541932)^2 = -5.88373(2\theta - 32.4212793573004)$$

Figure 2 shows the auxiliary graph of the second degree that causes the values close to the inverse equation of the fifth degree to converge.

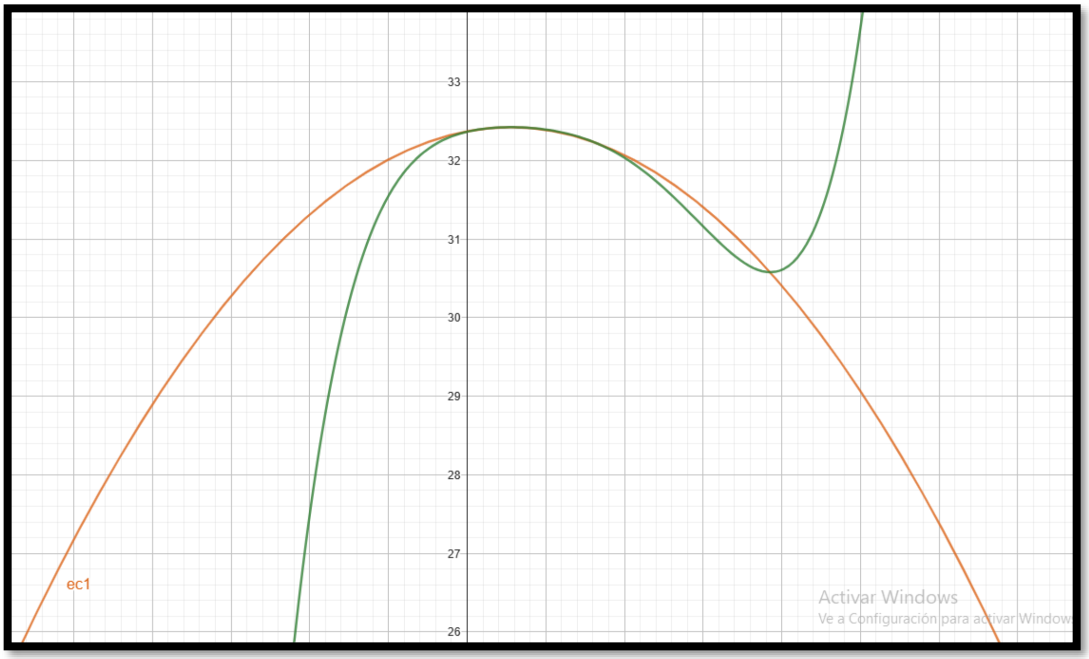

Figure S2 section graph of fifth degree equation and its second degree auxiliary.

In this way, the philosophy used to find the inverse function of a fifth degree equation in a given interval has been shown. In this work it can also be seen that the method is applicable to equations of greater or lesser degree and even to other types of functions.
